# Analysis of AMB-FUBINACA Biotransformation Pathways in Human Liver Microsome and Zebrafish Systems by Liquid Chromatography-High Resolution Mass Spectrometry

**DOI:** 10.3389/fchem.2019.00240

**Published:** 2019-04-16

**Authors:** Duo-qi Xu, Wen-fang Zhang, Jing Li, Ji-fen Wang, Shi-yang Qin, Jiang-hai Lu

**Affiliations:** ^1^China University of Political Science and Law, Beijing, China; ^2^Key Laboratory of Forensic Toxicology, Ministry of Public Security, Beijing, China; ^3^Drug Intelligence and Forensic Center of Ministry of Public Security, Beijing, China; ^4^College of Criminal Science and Technology, People‘s Public Security University of China, Beijing, China; ^5^Drug and Food Anti Doping Laboratory, China Anti-Doping Agency, Beijing, China

**Keywords:** AMB-FUBINACA, synthetic cannabinoids, HR-MS, human liver microsome, metabolism, zebrafish

## Abstract

In this study, the metabolic profiles of a new illicit drug AMB-FUBINACA were investigated using both human liver microsome and zebrafish models. Liquid chromatography Q Extractive HF Hybrid Quadrupole-Orbitrap mass spectrometry (LC-QE-HF-MS) was employed to analyze the metabolic sites and pathways. AMB-FUBINACA was added to the *in vitro* liver microsome incubation model to simulate the metabolic processes in human body. The results showed that a total of 17 metabolites were generated in the human liver microsome model; the main metabolic pathways of the phase I metabolism included ester hydrolysis, methylation, ester hydrolysis combined with decarboxylation, hydroxylation, ester hydrolysis combined with indazole ring hydroxylation, etc. while glucuronidation served as the main metabolic pathway of the phase II metabolism. The zebrafish system produced a similar result with 16 of the same 17 metabolites identified. The phase I metabolites M3.1 (ester hydrolysis), M1.2 (alkyl chain hydrolysis) and the phase II metabolite M3.2 (M3.1 glucuronide) were recommended to be the potential poisoning markers.

## Introduction

As of June 2018, 803 new psychoactive substances have been reported to the UNODC by 111 countries and territories, where new psychoactive substances of synthetic cannabinoids topped the list by covering 251 of them (accounting for 31.2%) (UNODC, 2018).

Synthetic cannabinoids (SCs) are a class of compounds similar in pharmacological and physiological effects to tetrahydrocannabinol (THC), the main active constituent of natural cannabis. By binding to the cannabinoid receptor CB1 and/or CB2, this class of substances can generate similar or even stronger physiological and pharmacological effects *in vivo* compared with THC. In March 2011, the Drug Enforcement Administration (DEA) first listed such drugs as Schedule I controlled substances^1^ Correspondingly, the legal measures were formulated in succession for the controlling consideration in many countries worldwide (EMCDDA, 2015). However, in order to evade legal sanctions (Langer et al., 2015; Qian et al., 2016), lawbreakers have been continuing to develop substitutes with similar pharmacological activities. Since naphthoylindoles as the first generation of SCs that appeared in 2006, they have evolved to eighth generation indazoles/indoleamides with reported coverage of more than 20 different structures^2^.

Most SCs are highly lipophilic and can induce extensive metabolism in the human body. As a result, it can be difficult to detect the parent drugs in the conventional biological samples, and the metabolite markers for monitoring the misuse of these drugs should be identified. Gas chromatography with mass spectrometry(GC-MS)and liquid chromatography with mass spectrometry(LC-MS)techniques are the most frequently used instrumentations for detecting synthetic cannabinoids. In aspect of identifying SC chemical structures, GC-MS has its limitation in detecting SC metabolites due to their high polarity and low volatility (Hasegawa et al., 2015a,b). In contrast, LC-MS is the preferred instrumentation for the analytical determination of non-volatile compounds, polar compounds, thermally unstable compounds and macromolecular compounds (Castaneto et al., 2015; Carlier et al., 2017a; Kavanagh et al., 2017).

**Figure 1:**
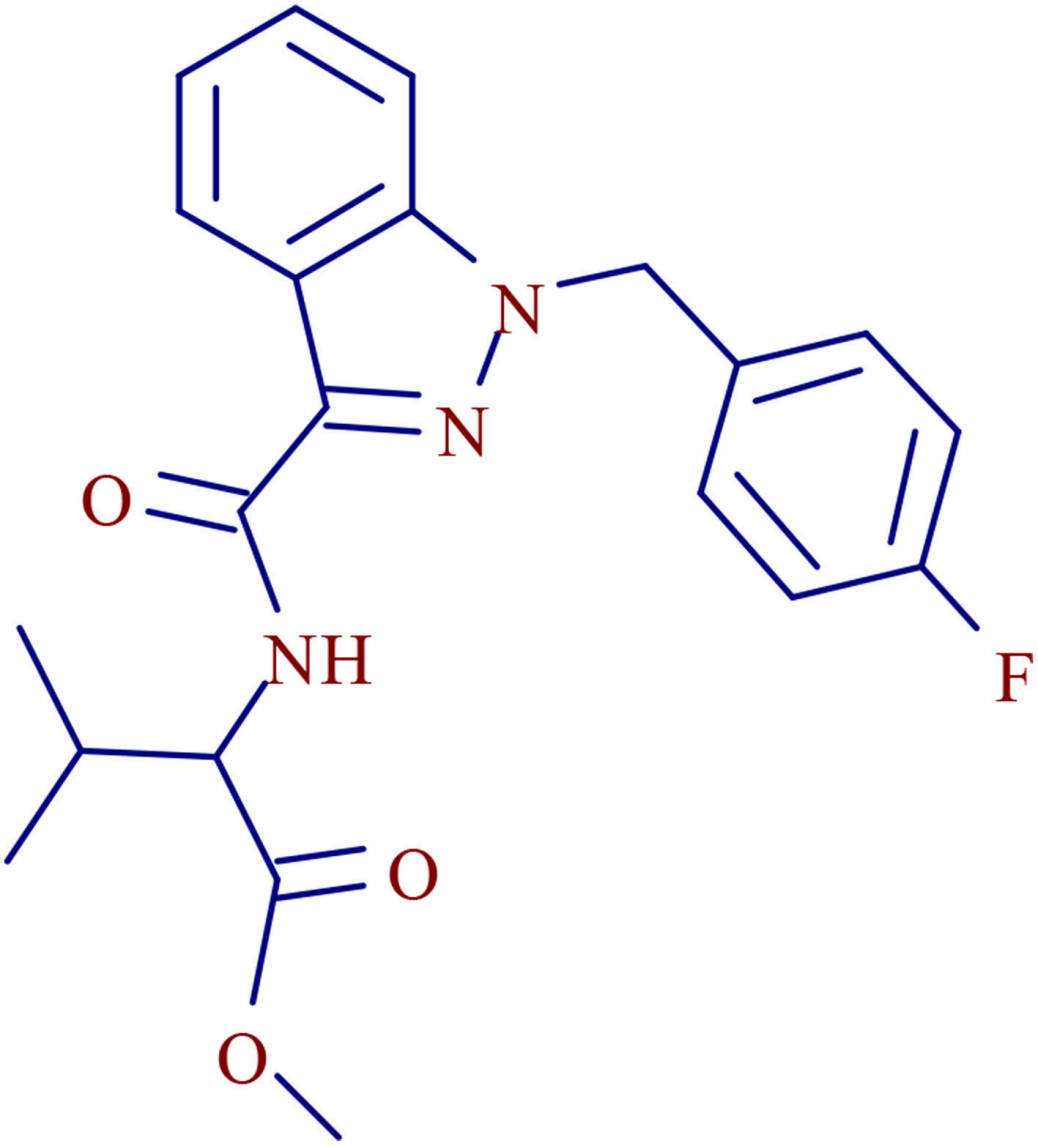
Chemical structure of AMB-FUBINACA.

3- methyl-2-[1- (4-fluorobenzyl) indazole-3-formamido] butyl butyrate (Figure 1), a AB-FUBINACA derivative, is a new psychoactive substance of SCs in the indazole-3-carboxamide family (Carlier et al., 2017b). It was first detected in the confiscated drugs in 2015 and has become one of the most popular SCs in recent years (Akamatsu and Yoshida, 2016; Qian et al., 2018a,b). AMB-FUBINACA-related cases were first reported in the “Zombie” Outbreak in New York in 2016 (Anghelescu, 2017). In June 2018, AMB-FUBINACA was included in the legal supervision list in China.

Due to the relatively short history of indoleamides and the lack in relevant international studies on such SCs, the investigation on the *in vivo* bioconversion and related metabolic pathways, in particular, of such drugs still remains in the primary stage. Based on the documentation of related literature, the main metabolic pathways of the newer generation SCs involve terminal amide and ester hydrolysis as well as hydroxylation combined with glucuronidation (Kavanagh et al., 2017).

Zebrafish is an important pharmaceutical tool for early drug discovery in recent years, and has been widely used in the process of screening and toxicity of drugs (Goldsmish, 2004; Jones et al., 2008; Sukardi et al., 2011). Studies have shown that zebrafish expresses a variety of drug metabolic enzymes, such as phase I metabolic enzyme cytochrome P450 (cytochrome P450, CYP450), and phase II metabolic enzyme uridine diphospho-glucuronosyl transferase (UGT) and sulfo-transferase (SULT), and so on, which are highly similar to the mammalian metabolic enzyme system (Bresolin et al., 2005; Valim Brigante et al., 2018). Zebrafish, representing a useful model for the study of human diseases with high throughput potential and efficacy, can be used to test the efficacy of neuroactive compounds (Bruni et al., 2016). Although the initial toxicity test of THC on zebrafish was conducted in 1975 (Thomas, 1975), up to now, there have been only a limited number of studies to use zebrafish as a model study drugs of abuse (Achenbach et al., 2018). In the current study, an *in vivo* metabolic animal model of zebrafish was established to verify themetabolic pathways of AMB-FUBINACA obtained from human liver microsomal experiment *in vitro*.

## Materials and Methods

### Instruments and Materials

AMB-FUBINACA (C_21_H_22_FN_3_O_3_: m/z, 384.17180; ≥ 98%) reference substance was obtained from the Key Laboratory of Forensic Toxicology, Ministry of Public Security; male and female liver microsomes (pHLM; protein concentration, 20 mg mL^−1^)was purchased from Beijing iPhase Biosciences (Beijing)Co., Ltd.; chromatographically pure acetonitrile, methanol, formic acid were purchased from Merck & Co., Inc.; ultrapure water prepared by Milli-Q Advantage A10 automatic distilled water machine which were purchased from Merck & Co., Inc.; NADPH regeneration system(1 mg mL^−1^ liver microsome mixture, 3.3 mM Mg^2+^, 1.3 mM NADP^+^, 3.3 mM Glucose-6-phosphate, 0.4 U mL^−1^ Glucose-6-phosphate dehydrogenase)was purchased from Beijing iPhase Biosciences (Beijing)Co., Ltd.; Uridine diphos phate glucuronic acid trisodium salt (UDPGA) was obtained from Rild Research Institute for Liver Diseases (Shanghai) Co., Ltd.; Zebrafish was obtained from Beijing University of Technology; SPE-Pak^@^Vac PSA Extraction column were purchased from Waters Co., Inc.

### LC and MS Conditions

The ultra high pressure LC system was connected to the Q Exactive HF MS (Thermo Fisher Scientific,USA) configured with HESI in series. The column was Thermo ®Hypersil GOLD column (50 × 2.1 mm, 1.9 μm); a 13 min gradient elution was developed with mobile phase A (5 mM ammonium formate, 0.1% (v/v) formic acid/water) and mobile phase B [0.1% (v/v) formic acid/ acetonitrile]. The elution procedure was carried out as follows: 5% B for 0.0–0.5min; 5–95% B for 0.5–6.5 min; maintaining 95% B for 6.5–11.5 min; 5%B for 11.5–11.6 min; maintaining 5% B for 11.6–13.0 min with the flow velocity at 0.3 mL·min^−1^. The column temperature was set at 30°C and the injection volume was 5 μL.

The instrument was calibrated under the positive ion mode prior to use. The conditions of ion source were set as follows: ion transfer capillary temperature, 320°C; auxiliary gas heating temperature, 350°C; sheath gas flow velocity, 40 AUs; auxiliary gas flow velocity, 10 AUs; spray voltage, 3.80 kV; S-lens RF level, 50.0. Under the MS positive ion full-scan mode, the parent ions of metabolites with specific mass-to-charge ratio were selected as the targets for secondary MS analysis. The exact molecular weight of the metabolites was calculated using the software Mass Frontier version 2.0 (Thermo).

The parameters of the full-scan data acquisition were set as follows: resolution, 60,000; automatic gain control (AGC), 3.0 × 10^6^; maximum injection time (IT), 50 ms; scan range, 100~1,000 m/z. The secondary parameters of the target ion were set as follows: resolution, 30,000; target AGC, 1.0 × 10^5^; maximum IT, 50 ms; isolation window, 1.0 m/z; normalized collision energy, 25, 35, and 45.

### Human Liver Microsome Study

AMB-FUBINACA was dissolved in methanol to prepare a solution at the concentration of 5 mmol·L^−1^, followed by adding 1 μL of it to the freshly prepared system solution to achieve the final system volume of 200 μL (regeneration system solution 12 μL+buffer solution 178 μL+liver microsome 10 μL). The reaction was initiated by the addition of a regeneration system and the mixture was then incubated for 1 h at 37°C. Then, UDPGA was added and incubated for another half an hour. After cooling to terminate the reaction 200 μL of acetonitrile was added, followed by centrifugation at 13,000 × g for 10 min; 100 μL of the supernatant was pipetted to the glass sample bottle after membrane filtering for subsequent instrument analysis. The system control and data acquisition for the sample was conducted with the software Trance Finder 4.1 General Quan version. While the blank solution, the incubated reaction system solution without target drug, the reaction system solution without liver microsomes and the reaction system solution without regeneration system were also analyzed as the controls.

### Zebrafish

**Figure 2:**
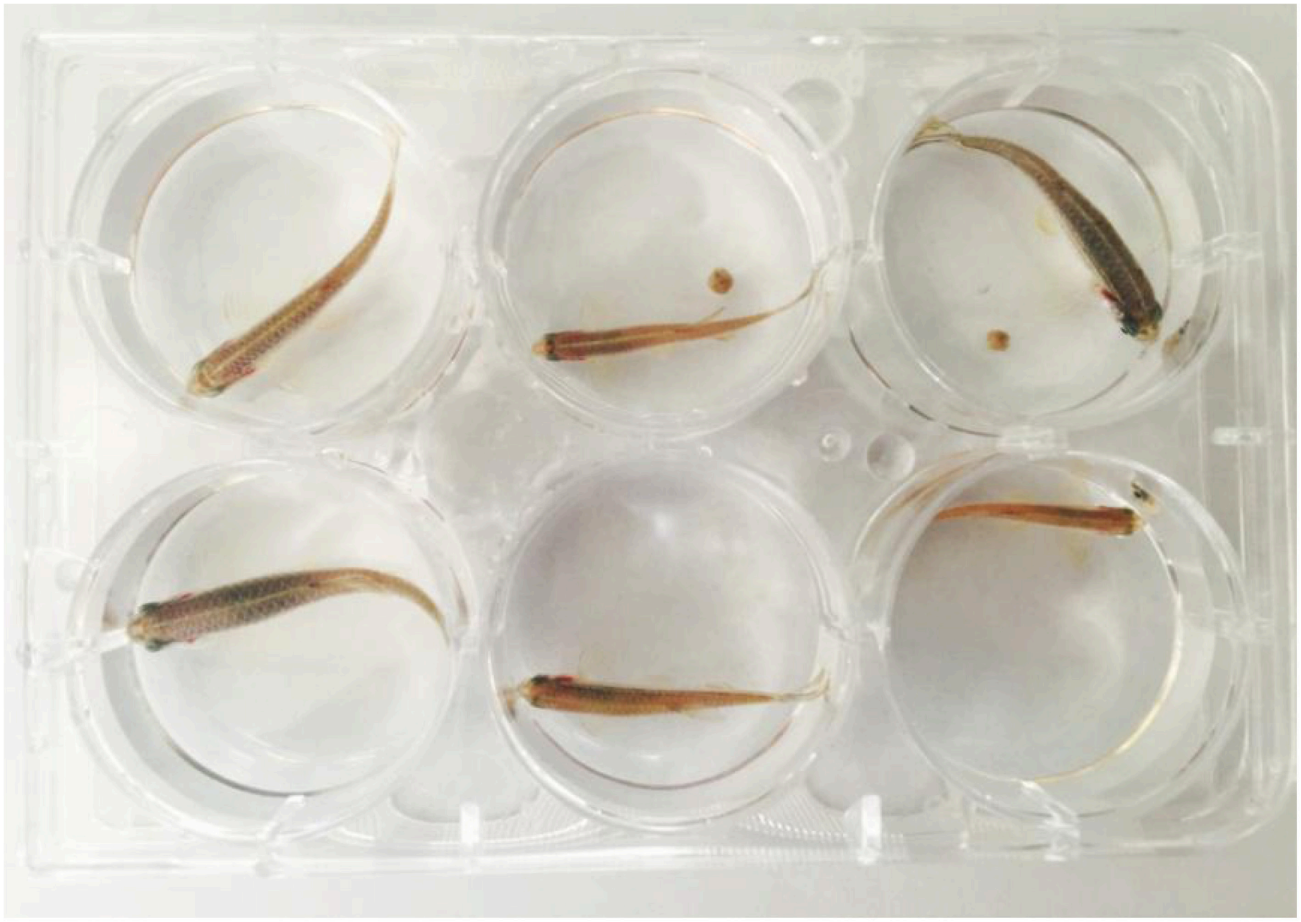
The zebrafish experiment groups with AMB-FUBINACA intake.

Adult male and female zebrafish (6–10 months; 0.8–1.2 g) were randomly divided into 4 groups with 3 fishes in each group. One group was the blank control group, and the other three groups were the experimental groups (Figure 2).

After exposing to 0.1, 0.5, and 1 μg/mL of AMB-FUBINACA in solution (24°C) for 24 h, zebrafish samples were removed, followed by cleaning with water and being euthanized. The zebrafish were homogenized with a ball mill, and the samples were loaded onto a PSA extraction column (1 mL), which had been conditioned with methanol (1 mL) and water (1 mL). The column was washed with acetonitrile (1 mL). The eluent was dried by evaporation at 60°C under a stream of nitrogen. The residue was reconstituted in the 100 μL flow phase composed of acetronile, and 10 μL of the reconstituted solution was injected for LC-QE HF analysis (Xu et al., 2016).

This study was carried out in accordance with the regulations and guidelines on using animals for scientific research purposes, the Animal Ethics Committee at China University of Political Science and Law.

## Results and Discussion

**Figure 3:**
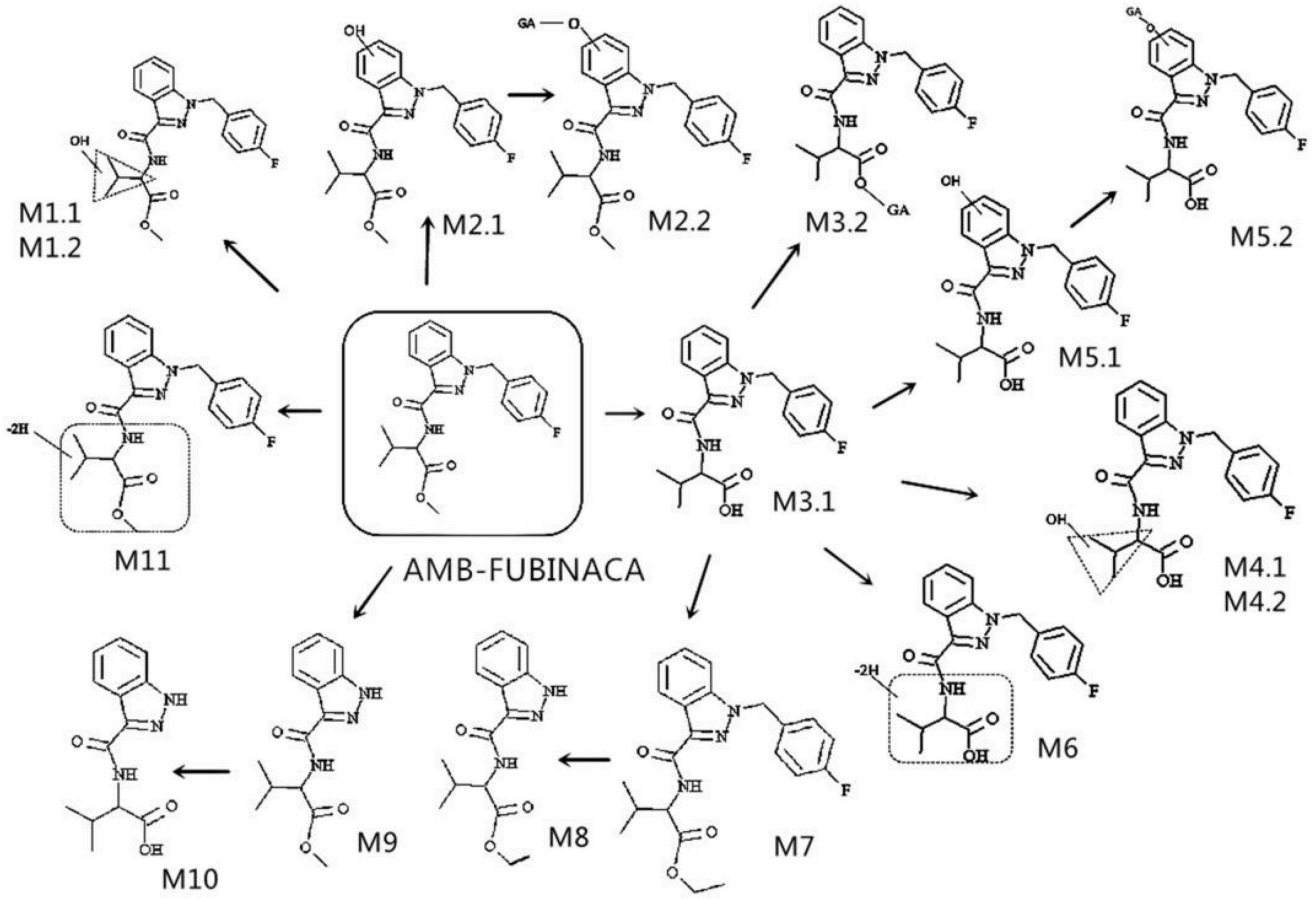
Proposed metabolic pathways of AMB-FUBINACA in human liver microsomes.

The characteristic fragment ions and the fragmentation pathways of AMB-FUBINACA parent structure were analyzed carefully. As shown in Figure 3, the core structure of acronym FUBINACA is composed of the fluorobenzyl substituent and indazole-formamido. Our experiment demonstrated that the amide bond was most susceptible to cleavage, thus forming the fragment ion at m/z 253.0772 (C_15_H_10_FN_2_O^+^), and the cleavage between the indazole ring and fluorobenzyl produced the fluorobenzyl ion (C_7_H_6_F^+^) at m/z 109.0448. Thus, the analysis of the structural characteristics of AMB-FUBINACA is helpful to the structural identification of its metabolites.

A total of 17 *in vitro* metabolites of AMB-FUBINACA were identified in the human liver microsome system by analyzing their accurate molecular masses and ddms2 spectra. The retention time of AMB-FUBINACA was determined to be 6.09 min, while the retention time of the 17 metabolites ranged from 4.04 to 6.43 min, where the mass errors were within 2.0 ppm (Table 1). These metabolites included 2 alkyl chain hydroxylated metabolites (M1.1, M1.2), 1 indazole ring hydroxylated metabolite (M2.1), 1 indazole ring hydroxylated metabolite combined with glucuronidation (M2.2), 1 ester hydrolysis metabolite (M3.1), 1 ester hydrolysis metabolite combined with glucuronidation (M3.2), 2 ester hydrolysis metabolites combined with alkyl chain hydroxylation (M4.1, M4.2), 2 ester hydrolysis metabolites combined with indazole ring hydroxylation (M5.1, M5.2), 1 ester hydrolysis metabolite combined with dehydroxylation (M6), 1 ester hydrolysis metabolite combined with ethylation (M7), 1 ester hydrolysis, ethylated metabolite combined with fluorobenyl loss (M8), 1 metabolite with fluorobenyl loss (M9), 1 ester hydrolysis metabolite combined with fluorobenyl loss (M10), and 2 metabolites with dehydroxylation (M11.1,11.2).

**Table 1:** Identification of AMB-FUBINACA metabolites with human liver microsomes.

**Peak ID**	**Metabolic reaction**	**RT/ min**	**Accurate mass(m/z)**	**Mass error**	**Chemical formula**	**Fragment ion (m/z)**
AMB- FUBINACA		6.09	384.1718	−0.2	C_21_H_22_FN_3_O_3_	109, 253, 324
M1.1	Hydroxylation (butane moiety)	5.21	400.1667	−0.2	C_21_H_22_FN_3_O_4_	109, 253, 382
M1.2	Hydroxylation (butane moiety)	5.35	400.1667	−0.2	C_21_H_22_FN_3_O_4_	109, 253, 382
M2.1	Hydroxylation (indazole)	5.69	400.1667	0.3	C_21_H_22_FN_3_O_4_	109, 269, 340
M2.2	Hydroxylation+ glucuronidation	4.67	576.1988	−1.2	C_27_H_30_FN_3_O_10_	109, 269, 445
M3.1	Ester hydrolysis	5.44	370.1561	−0.2	C_20_H_20_FN_3_O_3_	109, 253, 271
M3.2	Ester hydrolysis+ glucuronidation	4.73	546.1882	0.6	C_26_H_28_FN_3_O_9_	109, 253, 324
M4.1	Ester hydrolysis+ Hydroxylation (butane moiety)	4.74	386.1510	0.5	C_20_H_20_FN_3_O_4_	109, 253, 271
M4.2	Ester hydrolysis+ Hydroxylation (butane moiety)	4.81	386.1510	0.5	C_20_H_20_FN_3_O_4_	109, 253, 271
M5.1	Ester hydrolysis+ Hydroxylation (indazole)	5.00	386.1510	1.0	C_20_H_20_FN_3_O_4_	109, 269, 340
M5.2	Ester hydrolysis+ Hydroxylation+ glucuronidation	4.09	562.1831	−0.7	C_26_H_28_FN_3_O_10_	109, 269, 340
M6	Ester hydrolysis+ dehydrogenation	5.42	368.1405	0.4	C_20_H_18_FN_3_O_3_	109, 253, 271
M7	Ester hydrolysis+ ethylation	6.43	398.1874	−0.6	C_22_H_24_FN_3_O_3_	109, 253, 324
M8	Ester hydrolysis+ ethylation+ fluorobenzyl loss	5.15	290.1499	−1.9	C_15_H_19_N_3_O_3_	145, 163, 216
M9	Fluorobenzyl loss	4.84	276.1342	−1.2	C_14_H_17_N_3_O_3_	145, 163, 216
M10	Fluorobenzyl loss+ Ester hydrolysis	4.04	262.1186	−0.6	C_13_H_15_N_3_O_3_	145, 200, 216
M11.1	Dehydrogenation	5.43	382.1561	0.4	C_21_H_20_FN_3_O_3_	109, 253, 271
M11.2	Dehydrogenation	6.07	382.1561	−0.6	C_21_H_20_FN_3_O_3_	109, 253, 271

**Figure 4:**
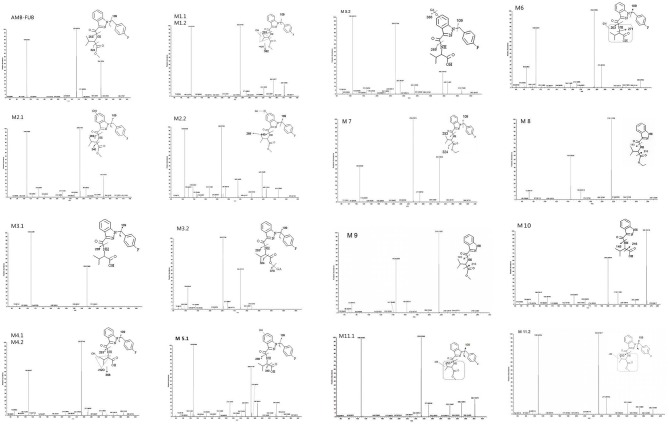
Product ion mass spectra and assigned fragmentation patterns for AMB-FUBINACA and all metabolites that were identified after human liver microsomes incubation.

The elemental compositions of protonated molecules of metabolites together with their theoretical m/z, mass errors, retention times, metabolic reactions, and the characteristics of product ions are listed in Table 1. Figure 3 shows the metabolic pathways of AMB-FUBINACA after *in vitro* liver microsome incubation. Figure 4 illustrates the characteristic fragment ions and fragmentation pathways of all metabolites.

### Metabolite of the M1 Group (Hydroxylation at Butane Moiety)

M1.1 and M1.2, the only two metabolites of this group, were detected in zebrafish samples associated with AMB-FUBINACA intake and in the human liver microsome system. The resulting protonated molecules fragmented in three ways, namely with loss of water (m/z 382), methanol (m/z 368), or a methylated carboxyl moiety (m/z 340), Fragmentation of the amide group afforded the m/z 253 ion.

### Metabolite of the M2 Group (Hydroxylation at Indazole)

The fragmentation of M2.2 included the elimination of the glucuronic acid moiety (M2.1). The fragmentation of M2.1 and M2.2, are similar to that of M1, with characteristic ions being generated by the loss of water (m/z 382) or the methylated carboxyl moiety (m/z 340). This fragmentation pathway exhibits carboxylation of a butane moiety. The spectra of the metabolites M2.1 and M2.2 were found to contain the fragment ions at m/z 269 and 109, suggesting that the hydroxyl group was located on the indazole moiety.

### Metabolite of the M3 Group (Ester Hydrolysis and Glucuronidation)

The metabolites of the M3 group were the most abundant in all zebrafish samples associated with AMB-FUBINACA intake. The glucuronidated metabolite M3.2 underwent a significant in-source fragmentation, forming the protonated M3.1 molecule which is common to glucuronidyl esters. Two product ions at m/z 324 and 253 in the spectra of metabolites M3.1 and M3.2 were formed by cleavage of the indazole side chains, indicating that the parent structure was not modified except for the terminal methyl ester. As the most important metabolites, ester hydrolysis metabolites *M3 group* can be used as potential poisoning markers for this drug.

### Metabolite of the M4 and M5 Groups (Ester Hydrolysis With Monohydroxylation and Glucuronidation)

Of the three monohydroxylated metabolites (M4.1, M4.2, and M5.1), the metabolite M4.2 with the hydroxyl group located on butane moiety was one of the most abundant metabolites in liver microsome experiment model and zebrafish experiment model. Upon fragmentation, the metabolites M4.1 and M4.2 initially dehydrated, generating a product ion at m/z 368 followed by the amide bond cleavage (m/z 253).The intense group at m/z 386 in the spectrum of glucuronide M5.2 corresponds to a protonated M5.1 molecule. The spectra of the minor metabolites M5.1 and M5.2 were found to contain the fragment ion at m/z 269, suggesting that the hydroxyl group was located on the indazole moiety. However, M5.1 was minor in samples associated with AMB-FUBINACA intake, Unfortunately, we were unable to detect the products of M5.2 in zebrafish samples.

### Metabolite of the M6 Group (Ester Hydrolysis and Dehydrogenation)

The fragmentation of M6, the only metabolite of this group, was detected in zebrafish samples associated with AMB-FUBINACA intake. Two product ions at m/z 324 and 253 are formed by cleavage of the indazole side chains, indicating the presence of modifications of the parent structure at the terminal methyl ester and/or one methyl group of the butane moiety.

### Metabolite of the M7 and M8 Groups (Ester Hydrolysis With Ethylation and Fluorobenzyl Loss)

The metabolites of M7 and M8 groups were found to be new compounds, which have never been reported before. Upon fragmentation, the metabolites M7 initially eliminated CH3CH2O (m/z 352), followed by a second elimination of carbon monoxide (m/z 324), with subsequent cleavage of amide bond (m/z 253). Two product ions at m/z 324 and 253 in the spectrum of M7 are formed by cleavage of the indazole side chains, indicating that the ethylation took place at the terminal hydroxyl. The fragmentation of M8, is similar to that for M7, with characteristic ions being generated by the loss of CH3CH2O and carbon monoxide. The metabolite of M7 was one of the most abundant metabolites in liver microsome experiment model and zebrafish experiment model, which was detected in all three experiment groups.

### Metabolites of the M9 Group (Fluorobenzyl Loss)

M9 generated ions at m/z 145 and 216, indicating the presence of unchanged indazole and butane moieties. The product ion at m/z 216 was formed by elimination of a free carboxyl group. The metabolite M9 was one of the most abundant metabolites in liver microsome experiment model and zebrafish experiment model, which was detected in all three experiment groups.

### Metabolites of the M10 Group (Ester Hydrolysis With Fluorobenzyl Loss)

The fragmentation of M10, the only metabolite of this group, was similar to that for M9,with characteristic ions being generated by the loss of a free carboxyl group (m/z 216).The relative peak areas for metabolite M10 were significant in liver microsome experiment model. However, they were minor in the zebrafish experiment model (Table 2).

**Table 2:** Absolute peak areas of AMB-FUBINACA and its metabolites in both human liver microsome model and zebrafish model.

	**Human liver microsome model**		**Zebrafish model**		
**Metabolite**	**Male (rank)**	**Female (rank)**	**Group1 (rank)**	**Group2 (rank)**	**Group3 (rank)**
AMB-FUBINACA			2.04 E8(1)	1.18 E8(1)	1.87 E8(1)
M1.1	n.d.	4.75 E5(14)	2.23 E4(9)	n.d.	3.83 E4(11)
M1.2	4.21E7(1)	1.53 E7(3)	5.61 E5(6)	5.52 E5(5)	n.d.
M2.1	1.26 E6(12)	1.15 E6(8)	n.d.	n.d.	2.09 E6(8)
M2.2	1.41 E6(10)	5.28 E6(6)	n.d.	9.27 E4(10)	5.56 E6(4)
M3.1	3.04 E7(2)	7.21 E7(1)	1.60 E8(2)	1.00 E8(2)	1.34 E8(2)
M3.2	3.55 E6(8)	8.89 E5(9)	n.d.	1.40 E4(11)	5.50 E6(5)
M4.1	2.67 E6(9)	4.69 E6(7)	n.d.	4.02 E5(6)	n.d.
M4.2	1.37 E7(5)	5.79 E6(4)	n.d.	5.60 E5(4)	n.d.
M5.1	1.32 E5(16)	3.47 E5(15)	n.d.	3.64 E3(12)	n.d.
M5.2	1.32 E6(11)	8.19 E4(17)	n.d.	n.d.	n.d.
M6	1.85 E5(15)	5.45 E5(13)	5.56 E6(4)	n.d.	5.13 E6(6)
M7	2.73 E7(3)	3.98 E7(2)	5.98 E5(5)	1.81 E6(3)	1.16 E7(3)
M8	4.04 E6(7)	8.48 E5(10)	n.d.	1.65 E3(14)	3.33 E3(12)
M9	1.52 E7(4)	5.67 E6(5)	1.56 E5(7)	3.18 E5(7)	3.14 E6(7)
M10	8.48 E6(6)	6.40 E5(11)	n.d.	2.04 E5(9)	1.57 E5(10)
M11.1	5.64 E5(13)	5.56 E5(12)	1.00 E5(8)	2.51 E5(8)	4.32 E5(9)
M11.2	2.05 E5(14)	1.95 E5(16)	6.33 E5(3)	2.04 E3(13)	n.d.

*The area ranks given in parentheses; n.d. not detected*.

### Metabolites of the M11 Group (Dehydrogenation)

Upon fragmentation, the metabolites M11.1 and 11.2 initially eliminated CH3CH2O (m/z 350). Two product ions at m/z 322 and 253 were formed by cleavage of the indazole side chains, indicating the presence of modifications of the parent structure at one methyl group of the butane moiety.

### Blank Control Trail

The reaction system solution without the target drug, without liver microsomes and without the NADPH regeneration system were used as control. No metabolites were detected in the solution of the reaction system without liver microsomes or in the samples of the incubation reaction system without the target drug, which proved that these metabolites were produced by the introduction of microsomes. In the reaction system without the regeneration system, only one metabolite M3.1 was detected, indicating that NADPH reductive coenzyme was an indispensable auxiliary factor in drug biotransformation reaction.

### Detection of Metabolites in Zebrafish Experiment Model

Sixteen metabolites were detected in the zebrafish model. No metabolites were detected in zebrafish control group. The four most abundant AMB-FUBINACA metabolites, M1.2, M3.1, M7, and M3.2, were detected in liver microsomal samples. These compounds detected in the zebrafish model confirmed the results of the liver microsomal assay.

### Potential Poisoning Markers for This Drug

Based on the documentation of related literature, the main metabolic pathways of new generation SCs involve terminal amide and ester hydrolysis as well as hydroxylation combined with glucuronidation (Kavanagh et al., 2017). As the most abundant metabolites, ester hydrolysis metabolites M3 group plays a very important role in the identification of this drug. However, these metabolites can be formed from other synthetic cannabinoids as well-including AB-FUBINACA (Hsin-Hung Chen et al., 2016). Therefore, identification of these metabolites on their own is not sufficient to indicate AMB-FUBINACA intake. The alkyl chain hydroxylated metabolites (M1.2) contains all the structural characteristics of AMB-FUBINACA and is one of the most abundant metabolites. In summary, the metabolites M3 group in combination with M1.2 were identified as the potential poisoning markers of AMB-FUBINACA in this study.

## Conclusion

Metabolism of AMB-FUBINACA was investigated using both human liver microsome and zebrafish systems, where ultra high pressure LC-HR-MS was employed to analyze the metabolic sites and metabolic pathways. The results demonstrated that the zebrafish system produced a similar result with 16 of the same 17 metabolites identified. The phase I main metabolic pathways included ester hydrolysis, methylation, hydroxylation, ester hydrolysis combined with indazole ring hydroxylation, etc. Glucuronidation served as the main phase II metabolic pathway. As the most important metabolites, M3.1 (ester hydrolysis), M1.2 (alkyl chain hydrolysis), and M3.2 (glucuronidation of M3.1) can be used as potential poisoning markers for this drug. Owing to the accurate, simple and efficient advantages, the analytical method developed in this study provides a detection tool in clinical and court cases against the abuse of this new psychoactive substance.

## Ethics Statement

This study was carried out in accordance with the regulations and guidelines on using animals for scientific research purposes, the Animal Ethics Committee at China University of Political Science and Law.

## Author Contributions

The experiment was designed by DX and she completed the entire experiment. WZ, JLi, SQ, JW, and JLu they provided technical guidance for the experiment.

### Conflict of Interest Statement

The authors declare that the research was conducted in the absence of any commercial or financial relationships that could be construed as a potential conflict of interest.

## Footnotes

^1^US DEA Drug Schedule. https://www.dea.gov/drug-scheduling.

^2^Regulation on the Control of Narcotic Drugs and Psychotropic Drugs of 2015,Pub.L.No.27-2015.
